# Comparative Analysis of the Feasibility of Myocardial Blood Flow Index Versus CT-FFR in the Diagnosis of Suspected Coronary Artery Disease

**DOI:** 10.31083/j.rcm2508284

**Published:** 2024-08-12

**Authors:** Qing-feng Xiong, Xiao-rong Fu, Yi-ju Chen, Ya-bo Zheng, Liu Wang, Wen-sheng Zhang

**Affiliations:** ^1^Image Center, Hainan Enhance International Medical Center, 571437 Boao, Hainan, China; ^2^Image Center, Wuhan Asia Heart Hospital, 430022 Wuhan, Hubei, China; ^3^Department of Pediatrics, Wuchang Hospital of Wuhan University of Science and Technology, 430062 Wuhan, Hubei, China

**Keywords:** coronary artery disease, CT-FFR, myocardial blood flow index, function assessment, diagnostic test

## Abstract

**Background:**

Using fluid dynamic modeling, noninvasive fractional flow reserve (FFR) derived from coronary computed tomography angiography (CCTA) data provides better anatomic and functional information than CCTA, with a high diagnostic and discriminatory value for diagnosing hemodynamically significant lesions. Myocardial blood flow index (MBFI) based on CCTA is a physiological parameter that reflects myocardial ischemia. Thus, exploring the relationship between computed tomography derived fractional flow reserve (CT-FFR) and MBFI could be clinically significant. This study aimed to investigate the relationship between CT-FFR and MBFI and to analyze the feasibility of MBFI differing from CT-FFR in diagnosing suspected coronary artery disease (CAD).

**Methods:**

Data from 61 patients (35 males, mean age: 59.2 ± 10.02 years) with suspected CAD were retrospectively analyzed, including the imaging data of CCTA, CT-FFR, and data of invasive coronary angiography performed within one week after hospitalization. CT-FFR and MBFI were calculated, and the correlation between MBFI or CT-FFR and invasive coronary angiography (ICA) was evaluated. Using ICA (value ≥0.70) as the gold standard and determining the optimal cutoff value via a diagnostic test, the diagnostic performance of MBFI or CT-FFR was evaluated.

**Results:**

MBFI and CT-FFR were negatively correlated with ICA (*r* = –0.3670 and –0.4922, *p* = 0.0036 and 0.0001, respectively). Using ICA (value of ≥0.70) the gold standard, the optimal cutoff value was 0.115 for MBFI, and the area under the curve (AUC) was 0.833 (95% confidence interval [CI]: 0.716–0.916, Z = 5.357, *p* < 0.0001); using ICA (value of ≥0.70) the gold standard, the optimal cutoff value was 0.80 for CT-FFR, and the area under the curve (AUC) was 0.759 (95% CI: 0.632–0.859, Z = 3.665, *p* = 0.0002). No significant difference was observed between the AUCs of CT-FFR and MBFI (Z = 0.786, *p* = 0.4316).

**Conclusions:**

MBFI based on CCTA can be used to evaluate myocardial ischemia similar to CT-FFR in suspected CAD; however, it should be noted that CT-FFR is a functional index based on the anatomical stenosis of the coronary artery, whereas MBFI is a physiological index reflecting myocardial mass remodeling.

## 1. Background

Recently, there has been an increasing emphasis on evaluating the functionality of coronary lesions [1, 2]. Several studies have clarified the value of functional indicators for diagnosing coronary artery disease (CAD) accurately. For example, fractional flow reserve (FFR)-guided percutaneous coronary intervention can significantly improve the prognosis of patients [3], the myocardial microcirculation resistance index can allow the evaluation of the degree of damage to the myocardial microcirculation [4], and myocardial blood flow can assess the semiquantitative degree of ischemia in patients with CAD [5]. Although the above methods are effective, they involve expensive, invasive examinations, making them less conducive to clinical popularization [6, 7].

Regarding noninvasive functional methods, FFR derived from coronary computed tomography angiography (CCTA) data, which mimics FFR based on the pressure measurement of the invasive catheter, can play a significant role in functional evaluation to assess myocardial ischemia. It provides a one-stop assessment of the anatomical and functional information of CAD through a single examination, without additional imaging and the use of vasoactive drugs, making it a new hot topic in clinical research. However, there are shortcomings, as its accuracy relies on high-quality CCTA imaging. Currently, the inclusion criteria for various large-scale clinical studies are relatively strict. Among patients who have experienced myocardial infarction in the past or have undergone revascularization, there is limited clinical data related to computed tomography derived fractional flow reserve (CT-FFR), which limits its widespread clinical application.

Researchers have also highlighted that CT-FFR, which mimics FFR based on the pressure measurement of the invasive catheter, can play a significant role in functional evaluation to assess myocardial ischemia using the cutoff value of ≤0.80 [8]. It has been increasingly accepted by the academic community in evaluating the risk of suspected coronary heart disease patients and has gradually moved from experimental to clinical use.

Our previous study demonstrated that myocardial blood flow index (MBFI), a reflective index derived from CCTA without additional scanning time and radiation dose, can evaluate the risk of CAD in suspected patients [2]. In the present study, using invasive coronary angiography (ICA) as the gold standard for determining high-risk suspected CAD, the potential feasibility of MBFI was evaluated in comparison with CT-FFR.

## 2. Objectives

This study aimed to further explore the diagnostic value of MBFI in determining the risk of suspected CAD.

## 3. Patients and Methods

### 3.1 Clinical Protocols (Data Collection from Medical Records)

**Fig. 1.**
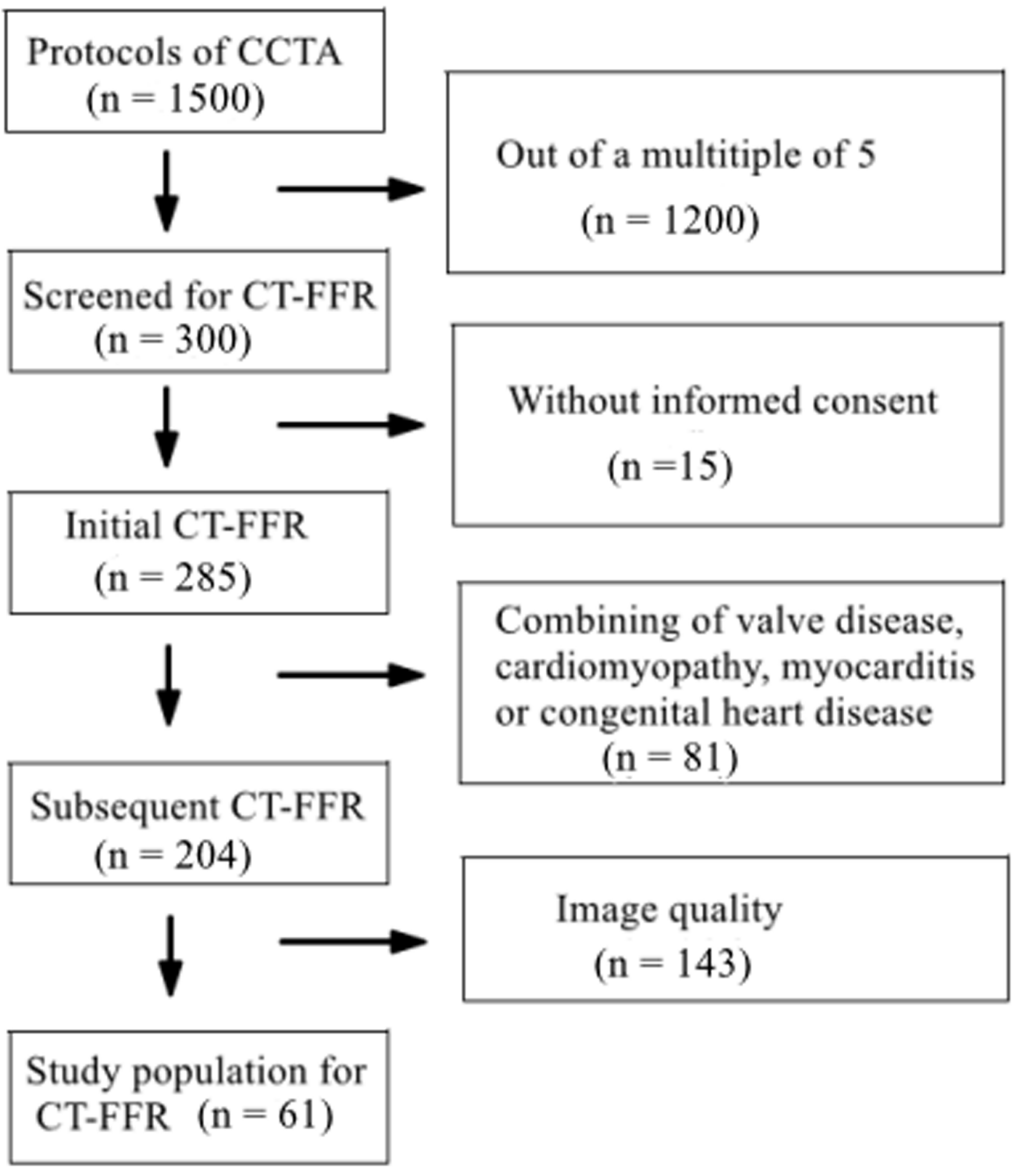
**Flowchart of patient selection**. Note: CCTA, coronary computed tomography angiography; CT-FFR, computed tomography derived fractional flow reserve.

Inclusion criteria: Clinical medical prospective data of 7500 patients suspected of angina pectoris (AP) (including stable AP, unstable AP, and atypical AP) [9] and who had undergone CCTA were continuously collected with continuous Arabic numeral numbering from March 2022 to October 2022. Patient data with a number in multiples of 5 were further analyzed via CT-FFR. A total of 61 cases were selected for the CT-FFR protocol (Fig. 1, Table 1). This study was approved by the ethics committee of the Hainan Enhance International Medical Center (No. 20220010). The study was implemented according to the standards of the Declaration of Helsinki (https://www.wma.net/policy/). All patients provided written informed consent.

**Table 1.** **Baseline characteristics (n = 61)**.

Characteristics		Total (n = 61)
Men/Women		35/26
Average age (years)		59.2 ± 10.02
History of risk factors		
	Hypertension ^a^	15
	Diabetes mellitus ^b^	10
	Dyslipidaemia ^c^	31
	Smoking (n)	13
Family history of CAD		6
Average heart rate (times/minute)		76 ± 10.1
Average diastolic blood pressure (mmHg)		82 ± 10.7
Average serum creatinine (µmol/L)		79
Average body mass index (kg/$m^{2}$)		24 ± 5.5
AP		
	Stable AP	46
	Unstable AP	15
Results of ICA		
	Normal	2
	One-vessel diameter stenosis	33
	Two-vessel diameter stenosis	17
	Three-vessel diameter stenosis	9
Average radiation dose		(3.06 ± 1.10) mSv

Values are mean ± SD, or frequencies (percentage). ^a^ Blood pressure ≥140/90 mmHg or treatment for hypertension. ^b^ Fasting blood glucose was above 7.0 mmol/L, or 2 hours after meal in glucose tolerance test was above 11.1 mmol/L. ^c^ Total cholesterol >180 mg/dL or treatment for hypercholesterolemia. Note: CAD, coronary artery disease; AP, angina pectoris; ICA, invasive coronary angiography.

Exclusion criteria: Patients with the following combined diseases were excluded: ST-elevation myocardial infarct, non-ST-elevation myocardial infarct, heart valve disease, cardiomyopathy, myocarditis, congenital heart disease, pulmonary hypertension, and emphysema. Patients with poor-quality CCTA scans, diffuse calcification in the coronary artery, and deep or diffuse myocardial bridge were also excluded.

### 3.2 CCTA Protocol

#### 3.2.1 Scanning Parameters

For the prospectively electrocardiogram-triggered CCTA (dual-source CT, SOMATOM Definition, Siemens Healthcare, Forchheim, Germany) protocol (Adaptive Sequential, Siemens Healthcare, Forchheim, Germany), the scanning parameters were set as follows using the CARE Dose 4D (Siemens Healthcare, Forchheim, Bavaria, Germany) fully automatic exposure control: Collimation, 2 × 64 × 0.6 mm with z-flying focal spot; gantry rotation time, 330 ms; pitch, 0.2–0.43; voltage/tube product, 100–120 kV. According to the weight of each patient, the tube current was fully automatically integrated. Image acquisition was triggered at 35%–75% of the R–R interval. The Hounsfield Unit (HU) value of the descending aorta was dynamically detected 10 s after the contrast agent (Bayer Schering Pharma, Berlin, Germany) injection using the contrast agent tracer technique. When the threshold was set at 120 HU, the trigger was initiated after 4 s. The personalized contrast agent dosage was calculated using the expected duration of the CT scan, and the total dosage was 50–70 mL (contrast agent: 370 mgI/mL iopromide, Ultravist, Bayer), followed by 30 mL of saline. The rate (3.8–4.2 mL/s) was adjusted according to the patient’s heart rate (HR), body mass index (BMI), and cardiac function, and if the brittleness of the patient’s blood vessel increased, an appropriate reduction in the injection rate was employed. CCTA image reconstruction was performed using a medium-smooth convolution kernel (B26f) at a slice thickness of 0.75 mm and increments of 0.3 mm using a single R–R interval reconstruction method. Image reconstruction was completed via 5% increments from 35% to 70% of the R–R interval. Subsequently, multiplanar reconstructions were generated, including maximum intensity projections and curved multiplanar reconstructions. The volume CT dose index and dose length product were used for CT automatic calculation.

#### 3.2.2 CT Image Reconstruction

Anonymized CCTA data were analyzed. The commercial syngo.via service platform (Siemens Medical Solutions, Erlangen, Germany; an offline work platform) was used. If several lesions in the same coronary artery existed, the more obvious lesion was selected and used for analysis. Using semiautomatic software, lesion severity was assessed, and the percent diameter reduction was calculated based on the lumen profile detected according to the minimum lumen and the corresponding reference diameter value obtained from the automatic variation trend of the total vessel. The degree of diameter narrowing was categorized as follows: normal, ≤50%, ≥50%–<70%, ≥70%–<99%, and complete occlusion.

#### 3.2.3 Image Quality Assessment

Two experts with 10 years of experience in CCTA evaluation conducted the image quality assessment. In case of disagreement, a consensus was reached through discussion. Images without artifacts or sharpness noise were selected [10].

### 3.3 MBFI

#### 3.3.1 Myocardial Blood Flow Model

**Fig. 2.**
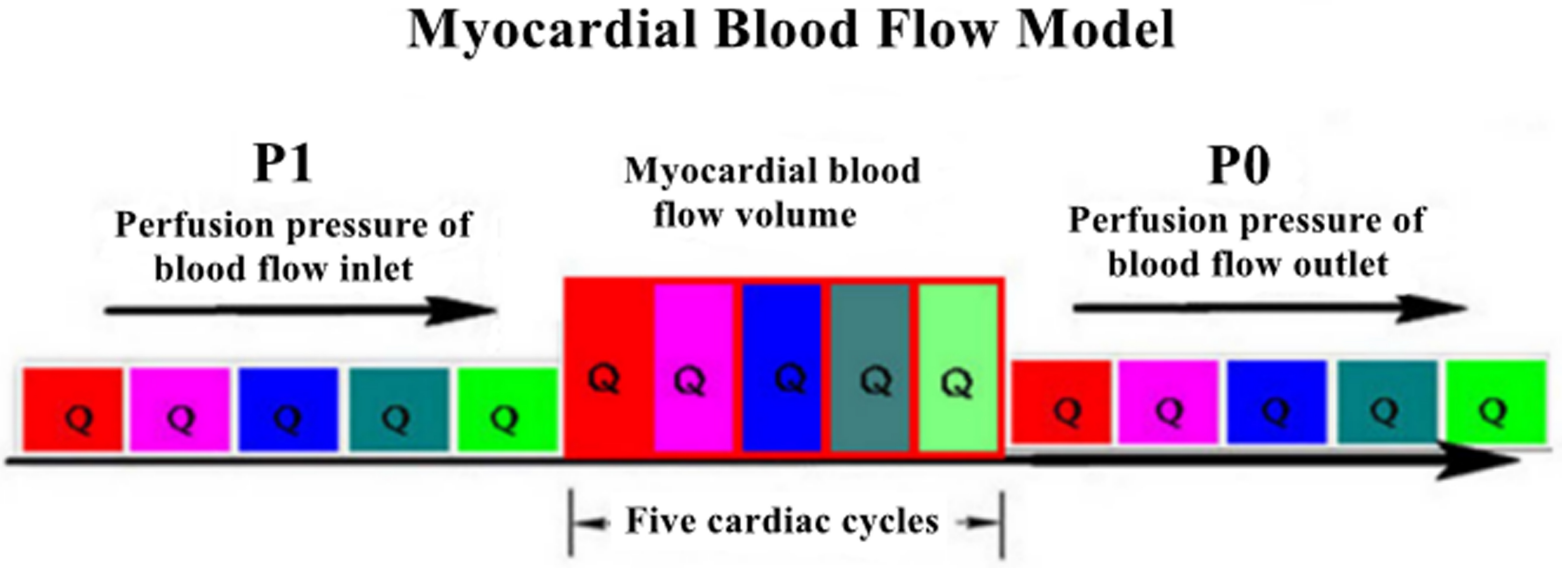
**Myocardial blood flow model**.

The myocardial blood flow model is based on a physical principle, i.e., the ratio of myocardial perfusion pressure to myocardial resistance (referred to as the myocardial blood flow) [11]. According to the lumped parameter network model [12], myocardial perfusion is a parallel characteristic impedance model [13], and the myocardial blood flow is primarily evaluated using myocardial blood perfusion pressure and myocardial microcirculation resistance (myocardial mass) [14]. Blood circulation in the heart comprises a series of pulses from the aorta to the venous end, and the entire process consists of approximately five cardiac cycles [15]. After five cardiac cycles, a certain volume of blood is distributed throughout the myocardium; hence, the myocardial blood flow to complete one cycle should include the blood flow of five cardiac cycles (Fig. 2). Furthermore, at rest, the right atrial pressure is usually 0 mmHg [16]; thus, the patient’s diastolic blood pressure (DP) can be considered as myocardial blood perfusion pressure. Therefore, the ratio of the DP multiplied by five cardiac cycles to the mass is called the myocardial blood flow. Since BMI [17], in addition to sex [18] and age [19], has been reported to be associated with myocardial blood flow [20], this study introduced a model of myocardial blood flow, which not only accounted for BMI and cardiac mass but also for sex and age to assess its clinical value in patients with chronic obstructive CAD [2, 11].

#### 3.3.2 Calculation of MBFI

The Omron arm sphygmomanometer (Omron U10K, Dalian, China) was used. The patient’s DP and HR were recorded in the sitting posture thrice, at intervals of 10 min. Subsequently, post-image processing software was used to measure cardiac mass (M). The cardiac cycle time (T) was measured as 60 s divided by HR in 1 min. The myocardial perfusion time (PT) is equivalent to 5 × T. Thus, MBFI = PT × DP/(M × BMI) [13, 15, 21]. For a 56-year-old female patient with an HR of 79 beats/min, DP of 80 mmHg, BMI of 27 kg/$m^{2}$, and M of 155 g, the patient’s MBFI is equal to (5 × 60/79) × 80/(155 × 27) – 0.015 × 1, which is 0.058.

### 3.4 CT-FFR Protocol

CT-FFR was analyzed in a blinded manner by two experienced observers at an independent core laboratory using a dedicated off-site software system (RuiXin-FFR, version 1.0, Raysight Medical, Shenzhen, China). The core laboratory indicated the location(s) of the wire-based FFR measurement(s) on a reconstructed coronary anatomy model [22].

### 3.5 Invasive Coronary Angiography (ICA)

ICA was performed according to the standard operating procedure used as the gold standard method for diagnosing CAD and severe CAD. At least six views of the left coronary arteries and two views of the right coronary arteries were projected. Two cardiologists with over 10 years of experience in cardiac catheterization, who were blinded to the results of CCTA, independently interpreted the angiograms. All coronary artery segments ≥1.5 mm in diameter were visually and quantitatively analyzed and condensed into 14 segments for comparison with CCTA data. Quantitative coronary angiography for the most severe stenosis was performed in all segments [23].

### 3.6 Statistical Analysis

Continuous variables are expressed as mean ± standard deviation, whereas categorical variables are expressed as frequency and percentage. The correlation between MBFI and ICA or CT-FFR and ICA was evaluated. Using ICA (value ≥0.70) as the gold standard and determining the optimal cutoff value via a diagnostic test, the diagnostic performance of MBFI or CT-FFR was evaluated. A receiver operating characteristic (ROC) curve was drawn for MBFI and CT-FFR. Using the DeLong test, the area under the curve (AUC) of MBFI was compared with that of CT-FFR. The chi-square test was used for enumeration-type data, and statistical significance was set at *p* < 0.05.

## 4. Results

**Fig. 3.**
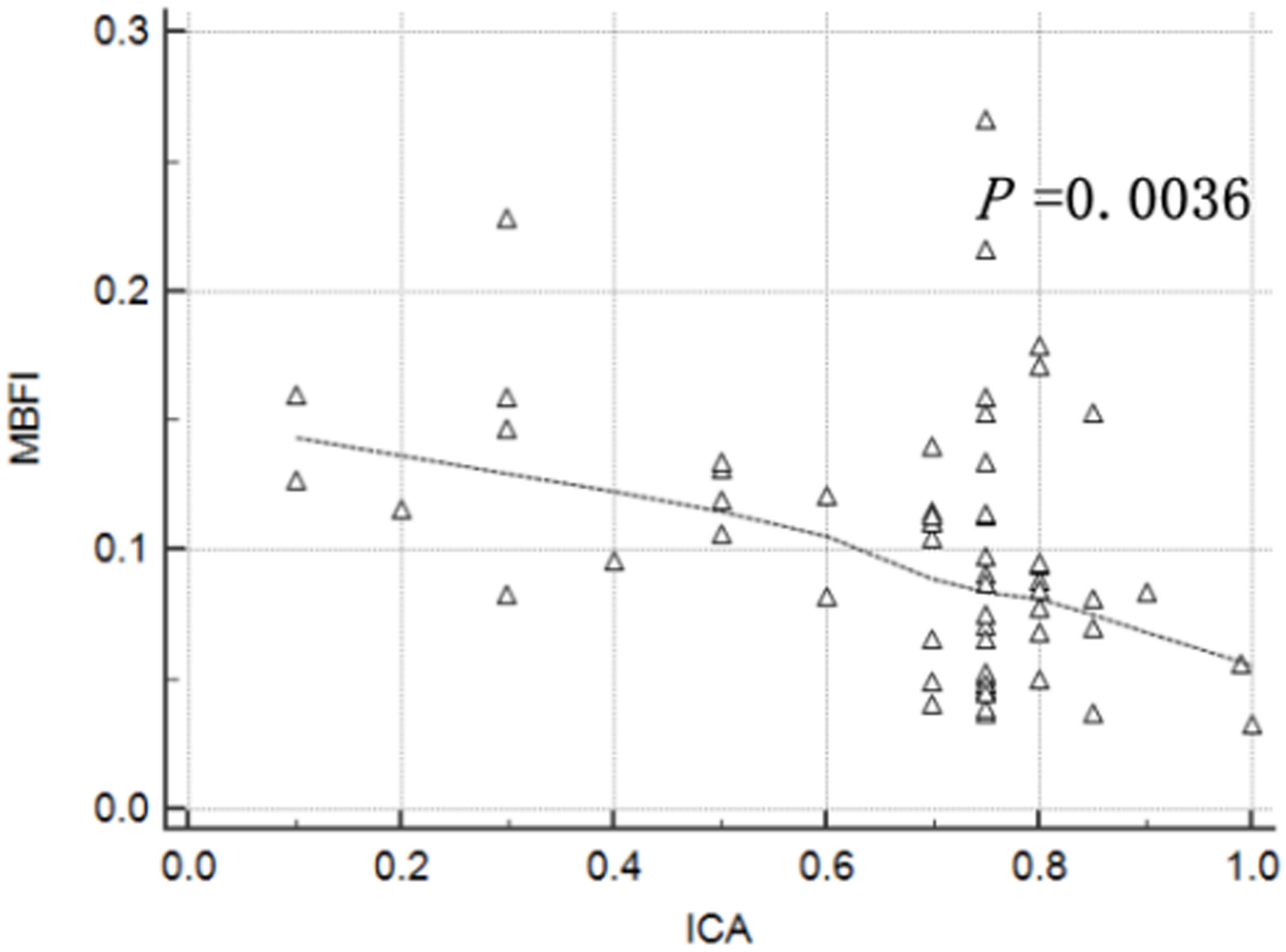
**Correlation between myocardial blood flow index (MBFI) and invasive coronary angiography (ICA)**.

**Fig. 4.**
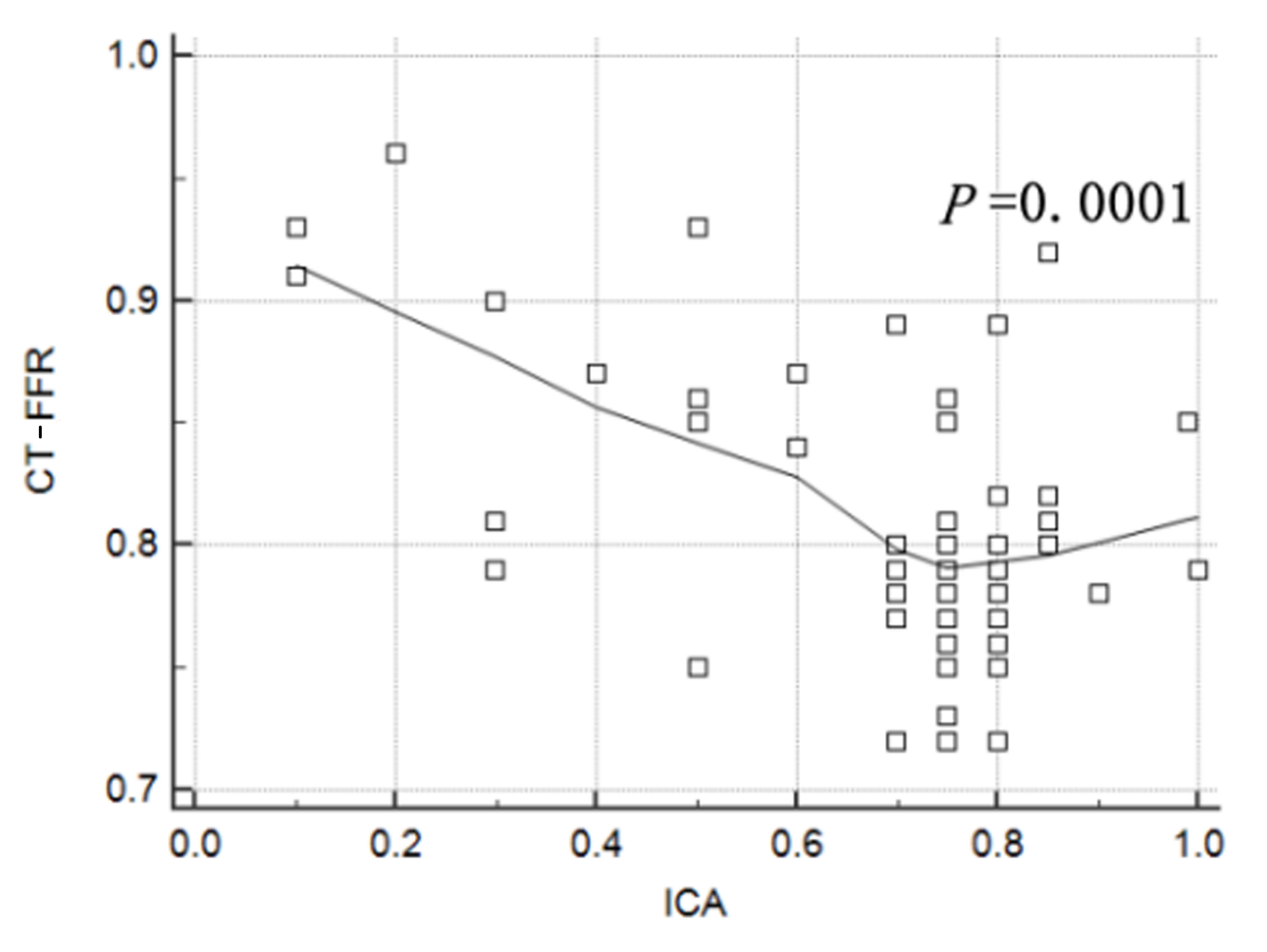
**Correlation between computed tomography derived fractional flow reserve (CT-FFR) and invasive coronary angiography (ICA)**.

MBFI and CT-FFR were negatively correlated with ICA (r = –0.3670 and –0.4922, *p* = 0.0036 and 0.0001, respectively; Figs. 3,4).

**Fig. 5.**
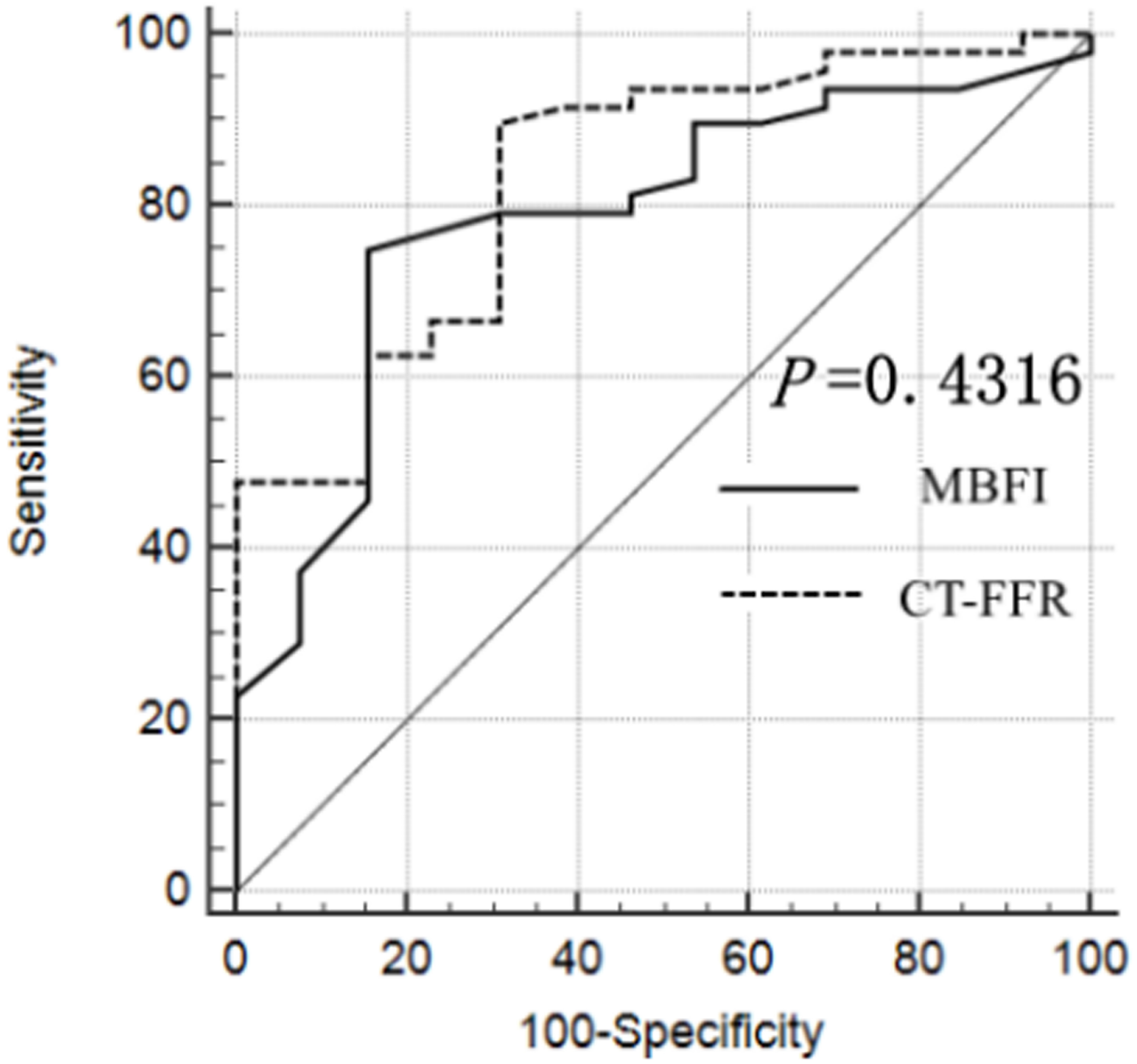
**Receiver operating characteristic (ROC) curve of myocardial blood flow index (MBFI) and computed tomography derived fractional flow reserve (CT-FFR) generated using invasive coronary angiography (ICA) as the gold standard**.

Using ICA (value ≥0.70) as the gold standard, the optimal cutoff value for MBFI was 0.115, with an AUC of 0.833 (95% confidence interval [CI]: 0.716–0.916, Z = 5.357, *p* < 0.0001). Using ICA (value ≥0.70) as the gold standard, the optimal cutoff value for CT-FFR was 0.80, with an AUC of 0.759 (95% CI: 0.632–0.859, Z = 3.665, *p* = 0.0002). No significant difference was observed between the AUCs of CT-FFR and MBFI (Z = 0.786, *p* = 0.4316; Fig. 5 and Table 2).

**Table 2.** **Receiver operating characteristic (ROC) analysis of myocardial blood flow index (MBFI) and computed tomography derived fractional flow reserve (CT-FFR)**.

	COV	Sensibility	Specificity	PPV	NPV	AUC	95% CI	*p* value
MBFI	0.115	80.85%	71.43%	90.48%	52.63%	0.833	0.716–0.916	<0.0001
CT-FFR	0.80	78.72%	92.86%	97.37%	56.52%	0.759	0.632–0.859	0.0002

Note: COV, cutoff value; PPV, positive predictive value; NPV, negative predictive value; AUC, area under the curve; CI, confidence interval.

We further evaluated the 6-month revascularization of the patients. Among the 61 patients diagnosed using CT-FFR and MBFI, 9 and 11 underwent revascularization, respectively, with no significant difference observed between the two groups ($\chi^{2}$ = 0.237, *p* = 0.6262).

## 5. Discussion

CCTA is a safe, accurate, and reliable noninvasive imaging method to diagnose and screen low- and medium-risk CAD [24, 25]. However, it does not allow functional evaluation of severe stenosis or suspected severe stenosis coronary lesions, which is vital for their treatment [26]. Similar to other functional methods [1, 2, 3, 4, 5], CT-FFR based on coronary artery anatomical stenosis has significantly improved patients’ prognoses [20].

CT myocardial perfusion imaging also belongs to the category of functional imaging. Traditional CT myocardial perfusion refers to continuous CT scanning during cardiac-specific phases using high-pressure injectors to inject iodine-containing contrast agents intravenously to obtain the time variation curve. The mathematical model of the curve is used for semi- or full-quantitative evaluation of the myocardial blood flow and then evaluation of coronary artery-related myocardial ischemia or microvascular angina [27]. After injecting an iodine-containing contrast agent, scanning during a fixed phase can obtain a fine contrast of the ischemic myocardium to the normal myocardium in the resting state. Adenosine stress perfusion can further obtain the compensatory ability of myocardial blood flow [28]. Therefore, a one-stop cardiac scanning protocol can evaluate the anatomical stenosis of coronary arteries and the coronary artery-related myocardial perfusion, which is helpful for detecting hemodynamically significant CAD. If the scanning protocol adds delayed scanning, it can predict myocardial viability [29]. It is not difficult to see that the shortcomings of CT perfusion perhaps include the complex protocol of CT scanning, the increased risk of myocardial infarction in patients with coronary artery disease during myocardial stress perfusion, increased radiation dose during delayed scanning, and prolonged overall scanning time, which imperceptibly increases patient discomfort. Compared with the traditional method of evaluating myocardial perfusion, MFBI has potential outstanding clinical application value. Our previous study demonstrated that MBFI, as a functional parameter, can be used to evaluate myocardial ischemia in obstructive CAD [11]. Since MBFI is closely correlated with myocardial remodeling, it can also be used to evaluate in-stent restenosis [2]. Therefore, elaborating on the differences in the functional features between CT-FFR and MBFI is necessary.

CT-FFR is based on the anatomical model of the coronary artery, combined with mathematical models of coronary artery physiology and physical laws of fluid dynamics, to obtain the results of simulated catheter method FFR, and is a highly effective noninvasive imaging method, and as a functional index, a CT-FFR value of ≤0.80 is used to determine whether coronary artery stenosis will lead to myocardial ischemia. The procedure mainly mimics the value of catheter FFR, which is feasible in clinical practice [30]. However, CCTA-FFR has certain limitations, such as high image quality requirements (which may increase additional radiation) and the need for additional computer post-processing analysis (which increases additional costs). Similar to CCTA-FFR, MBFI is also a noninvasive method derived from CCTA [2, 11], which can help address some of the limitations of CCTA-FFR.

Using ICA (≥0.70) as the standard, the optimal cutoff point for MBFI was 0.115 with a sensitivity of 80.85%, specificity of 71.43%, positive predictive value of 90.48%, negative predictive value of 52.63%, and accuracy of 78.69%, whereas the optimal cutoff point for CCTA-FFR was 0.80, and the corresponding values were 78.72%, 92.86%, 97.37%, 56.52%, and 81.97%, respectively. No difference in AUC was observed between MBFI and CT-FFR (Z = 0.786, *p* = 0.4316), and there was no difference between them in terms of accuracy (*p* > 0.05). Regarding the lower specificity of MBFI at 71.43% (compared to that of CCTA-FFR at 92.86%), the reason may be that myocardial remodeling had not yet begun or was not yet evident for lesions leading to hemodynamic abnormalities.

According to the literature, CT-FFR is significantly correlated with coronary artery anatomical stenosis, indicating that CT-FFR, as a functional parameter, is closely related to coronary artery anatomical stenosis [31]. In this study, CT-FFR was negatively correlated with ICA (*p* = 0.0001), and the more severe the anatomical stenosis of the coronary artery, the smaller the CCTA-FFR. Moreover, MBFI was also negatively correlated with ICA (*p* = 0.0036). Interestingly, as the degree of stenosis worsened, MBFI showed an increasing trend. This could be because CT-FFR was based on coronary artery anatomical stenosis, whereas MBFI was not only related to coronary artery anatomical stenosis but also to other factors such as CAD functional features and myocardial collateral circulation.

## 6. Limitations

This study had several limitations. First, the number of cases was relatively small as this was a single-center study; thus, a large sample size and multicenter research are warranted in the future. Second, the selected cases were suspected cases of CAD; however, there may be patients with coronary artery spasms or abnormal myocardial microcirculation function, resulting in inconsistent CT-FFR and MBFI results. Third, this study also needs to consider some confounding factors, such as sex differences in patients and the professional quality of the personnel using the technology.

## 7. Conclusions

First, in this study, MBFI is based on CCTA similar to CT-FFR, which is also derived from CT; Second, MBFI can be used to evaluate myocardial ischemia similar to CT-FFR in suspected CAD; Third, it should be noted that CT-FFR is a functional index based on the anatomical stenosis of the coronary artery, whereas MBFI is a physiological index reflecting myocardial mass remodeling.

## Data Availability

The datasets used and/or analyzed during the current study are available from the corresponding author on reasonable request.
